# Health and Social Care Professionals’ Experience of Psychological Safety Within Their Occupational Setting: A Thematic Synthesis Review

**DOI:** 10.3390/nursrep15040131

**Published:** 2025-04-14

**Authors:** Nicola Peddie, Josephine Hoegh, Gemma Rice, Shruti Shetty, Aoife Ure, Nicola Cogan

**Affiliations:** Department of Psychological Sciences and Health, University of Strathclyde, 40 Graham Hills Building, Glasgow G1 1QE, UKnicola.cogan@strath.ac.uk (N.C.)

**Keywords:** psychological safety, health and social care professionals, workplace culture, scoping review, thematic synthesis

## Abstract

**Objective:** Psychological safety (PS) is essential for health and social care professionals (HSCPs) working in high-stress environments. While much of the existing research focuses on PS within teams, less is known about HSCPs’ lived experiences across diverse health and social care settings. This scoping review aims to synthesise the qualitative literature on PS, identifying key barriers and enablers to its development in health and social care workplaces. **Methods:** A systematic search was conducted across MEDLINE, PsycINFO, Embase, CINAHL, Scopus, Web of Science, and the Cochrane Library, covering a 20-year period (2004–2024). Eligible studies included primary qualitative research exploring HSCPs’ experiences of PS. Screening and data extraction were managed using Rayyan. An inductive thematic synthesis approach was applied to identify key patterns in the data. **Results:** The review identified several enablers and barriers to PS. The main enablers included (1) feeling safe within the team and (2) personal factors, which encompassed professional skills, experience, social support, and self-care. Conversely, key barriers were identified: (1) the normalisation of traumatic incidents, (2) unsupportive team and management structures, (3) organisational constraints, and (4) a lack of knowledge and training on PS. **Conclusions:** Understanding the enablers and barriers of PS is critical for improving workplace culture, resilience, and wellbeing among HSCPs. These findings provide a foundation for future research and interventions aimed at strengthening PS at individual, team, and organisational levels across diverse health and social care settings. The results also offer valuable insights for informing policies and practice to ultimately enhance both staff wellbeing and patient care quality.

## 1. Introduction

Occupations within health and social care services, including nursing, medicine, social work, rehabilitation, welfare work, physiotherapy, occupational therapy, mental health support, and paramedicine, are essential to the functioning of society and often deeply rewarding [1]. These professions provide a sense of purpose, human connection, and the opportunity to make a meaningful difference in the lives of others. However, they are also widely recognised as among the most stressful of all occupations [2,3,4,5,6,7,8]. The combination of high emotional demands, exposure to trauma, heavy workloads, and management of complex patient needs creates a challenging environment that significantly impacts the health and wellbeing of HSCPs.

While HSCPs experience job satisfaction and fulfilment from helping others [9], the associated stress can contribute to serious health risks. The physical consequences of prolonged workplace stress can be severe, including an increased risk of cardiovascular diseases [10,11], musculoskeletal disorders [12], gastrointestinal problems [13], and metabolic disorders [14]. Additionally, HSCPs often report heightened susceptibility to mental health challenges, including burnout, anxiety, depression, chronic fatigue, compassion fatigue, moral injury, post-traumatic stress disorder (PTSD), and suicidal thoughts [15,16,17,18,19,20]. Among all professional occupations, HSCPs have the highest rates of suicide [21], with many facing a constant battle to manage the emotional and psychological toll of their work.

The COVID-19 pandemic has exacerbated these challenges, placing unprecedented stress on HSCPs and increasing the mental and physical burdens of their roles [22]. HSCPs working on the frontlines of COVID-19 care, for instance, faced compounded stress from higher patient loads, exposure to illness, and the emotional burden of witnessing suffering, leading to significant mental health crises among many [16,23]. As a result, there is a growing recognition of the need to understand and address factors that can help mitigate these risks, protect HSCPs’ health, and improve the overall work environment [24].

### 1.1. The Importance of Psychological Safety at Work

Psychological safety (PS) has emerged as a key factor in fostering a healthier, more effective work environment for HSCPs [25]. PS is foundational in ensuring that professionals feel supported and capable of managing workplace stress without fear of negative consequences. Originally described by Schein and Bennis [26] as an essential element of organisational development, PS was later defined by Kahn [27] as an individual’s sense of being able to express oneself without fear of harming one’s self-image, status, or career. In the context of team dynamics, Edmondson [28] characterised PS as “a shared belief that the team is safe for interpersonal risk-taking”.

PS is particularly important in the context of healthcare and social care settings, for HSCPs operate within a high-stakes environment where collaboration, communication, and trust are essential to providing safe and effective care. The sense of PS encompasses the ability to speak up about patient care issues, share concerns, and seek help when needed without fear of blame or retaliation. Such an environment is crucial for reducing medical errors, improving team collaboration, and ensuring patient safety [29,30,31]. At the same time, PS is vital for the emotional and psychological wellbeing of HSCPs themselves, helping them manage stress, prevent burnout, and foster post-traumatic growth [32,33].

Conversely, when psychological safety is embedded within the workplace culture, it promotes engagement, job satisfaction, and retention—factors that are increasingly important in the face of workforce shortages and high turnover. A workplace that promotes PS not only improves communication and teamwork but also supports HSCPs in navigating the stresses of their roles, thus enhancing both their individual and collective health outcomes [34].

### 1.2. The Neurophysiology of Psychological Safety

The neurophysiological foundation of PS is also gaining increasing attention, particularly through the lens of polyvagal theory (PVT) [35], which examines how stress impacts the nervous system. PVT explains how individuals respond to stress by activating different autonomic states: ventral vagal (VV), sympathetic activation (SA), and dorsal vagal (DV), which relate to PS [36]. Stressful work conditions, such as those faced by HSCPs, can lead to the activation of the body’s ‘fight or flight’ response (SA) or shutdown response (DV), both of which inhibit optimal functioning and increase vulnerability to burnout [37]. The prolonged exposure to such stress responses leads to an elevated cortisol level, which can cause chronic health issues, including weakened immune function, cardiovascular strain, and even digestive problems [35]. Understanding these neurophysiological mechanisms highlights the importance of fostering PS [38], as a supportive and trauma-informed environment can help regulate these stress responses and contribute to improved mental and physical health [39].

### 1.3. The Role of Psychological Safety in HSCPs’ Wellbeing

The wellbeing of HSCPs is not only vital for their own health but also for delivering safe, high-quality care to patients [40]. Despite its importance, many HSCPs report a lack of perceived PS in their workplaces, which prevents them from speaking up or addressing concerns, leading to potential errors and heightened stress. Fear of retribution, being ignored, or causing trouble often silences these voices [41]. This culture of silence can contribute to unsafe working conditions, reduce team effectiveness, and increase the likelihood of burnout, moral injury, and turnover among staff. However, research suggests that certain organisational factors can enable PS. These include a positive safety culture, continuous improvement efforts, cohesive team dynamics, ethical leadership, and change-orientated organisational practices [42]. When these elements are in place, HSCPs are more likely to feel safe, supported, and valued, leading to higher levels of engagement and improved job satisfaction, which can significantly improve their mental and physical wellbeing and contribute to safer patient care [43].

## 2. Focus of the Scoping Review

While much of the existing research on PS has examined its measurement and impact across various occupations [44,45,46], teams [42,43], and at the individual level [9,33,47], there is a growing body of research focused on understanding how HSCPs themselves experience PS in the workplace. This scoping review aims to synthesise the existing qualitative research on the barriers and enablers of PS from the perspective of HSCPs to better understand the factors that influence their ability to feel psychologically safe at work.

## 3. Objective of the Review

The objective of this scoping review is to identify, collate, and thematically synthesise qualitative research on the factors that enable or act as barriers to PS in health and social care settings. By capturing the lived experiences of HSCPs, the review will inform future interventions and research designed to enhance PS at individual, team, and organisational levels, ultimately improving HSCPs’ wellbeing and the quality of patient care.

## 4. Research Questions

- Primary Question

What are HSCPs’ experiences of PS within their workplaces?

- Secondary Questions

How is PS conceptualised in the research literature?

1. What are the characteristics of participants included in the sources of evidence identified?
2. What research designs underpin the literature on PS as experienced by HSCPs?
3. What factors are effective in facilitating (enablers) or reducing (barriers) PS among HSCPs?
4. What gaps exist in the literature regarding PS as experienced by HSCPs?

## 5. Method

The purpose of the scoping review method is to map a body of peer-reviewed research literature with the intention to illuminate key characteristics, terms, methods, findings, and relevant gaps to inform future research [48]. The Joanna Briggs Institute (JBI) methodology and checklist for conducting scoping reviews were followed [49]. In terms of reporting style, the Preferred Reporting Items for Systematic reviews and Meta-Analyses extension for Scoping Reviews checklist (PRISMA-ScR) was used [50]. The scoping review adhered to the ENTREQ statement and utilised thematic synthesis [51]. The scoping review protocol was prospectively registered with OSF (https://osf.io/preprints/psyarxiv/4u7hq (accessed on 20 May 2024)) and was published as Hoegh et al. [52].

## 6. Inclusion Criteria

### 6.1. Population

The study population included specific mention of ‘health and/or social care professional’ and their experiences of individual or group PS in their work setting. In studies where accounts of HSCPs’ own understandings of PS are included, their own use of terminology (e.g., feeling safe, social engagement) was acknowledged.

Only qualitative studies were included to capture the personal experiences and perspectives of HSCPs’ in terms of PS, as qualitative research enables a deeper exploration of underlying beliefs, concerns, and contextual factors that shape HSCPs’ sense of PS.

### 6.2. Context

The context was to explore research published worldwide.

### 6.3. Date of Publication

From March 2000 to February 2024. Databases were searched from 2000, as it was believed that the term “Psychological Safety” became popularised by the work of Edmondson [28] in the late 1990s.

### 6.4. Types of Evidence

Primary research, qualitative, or mixed methods research which included qualitative data.

### 6.5. Languages

English only (based on this being the primary language spoken within the context being researched).

## 7. Exclusion Criteria

Studies that did not include peer-reviewed primary qualitative data, such as quantitative studies, reviews, opinion texts and grey literature, were not sourced. Studies that include qualitative data but do not provide a sufficient proportion for meaningful analysis and inclusion in a thematic synthesis. If a significant proportion of the study population did not focus on HSCPs, then the paper was excluded.

## 8. Calibration

Prior to commencing the screening process, a calibration exercise was conducted between reviewers [53]. This consisted of selecting 10% of the papers for independent screening by each reviewer. A high level of agreement among reviewers was achieved (higher than 90%) [54,55].

## 9. Sources of Evidence

Two levels of screening were used to identify sources of evidence for inclusion in the scoping review: (a) study selection—review title and abstract, (b) study screening—review the full text. Data screening, charting and literature quality assessments were managed using Rayyan software (http://rayyan.qcri.org, (accessed on 20 May 2024)) [56] to sift, categorise, sort and store findings according to key issues and themes. Any articles identified as relevant based on the title and abstract were reviewed at the full-text level. A PRISMA flowchart was used to report the final number of the study selection process.

## 10. Search Strategy

The search strategy for this scoping review was designed to be as comprehensive as possible and developed with the help of an expert health librarian. It was peer-reviewed using the Peer Review of Electronic Search Strategies (PRESS) guidelines [57]. Following the JBI guidelines for conducting a scoping review, a three-step search strategy process was implemented:

1. Initial Search: The first step involved performing an initial search of two databases, namely MEDLINE (Ovid) and APA PsycInfo. Text words used in the titles and abstracts of relevant papers identified within this search were extracted and analysed alongside the index terms describing the articles.
2. Comprehensive Database Search: These terms were then used in step two, where a further search was conducted across all relevant databases: MEDLINE, APA PsycINFO, Embase, CINAHL, Scopus, Web of Science, and Cochrane Library.
3. Reference List Examination: Lastly, step three included examining the reference lists of articles included in the review to identify further relevant sources.

Grey literature was not included in the final review. 

The SPIDER (Sample, Phenomenon of Interest, Design, Evaluation, Research type) tool was utilised as the source for the search strategy [58]. This tool was used to define key elements of the review question and search strategy, ensuring a systematic approach to identifying relevant literature (see Table 1).

## 11. Data Extraction Process

Data were extracted, duplicates removed, and titles and abstracts were screened. Papers which met the inclusion criteria were retained for full-text screening by four independent reviewers who each screened 50% of the papers. If an abstract did not provide sufficient exclusion information, the article was retained for full-text screening. Two independent reviewers screened full papers using the inclusion and exclusion criteria, and any uncertainty was resolved through discussion. Data extracted from selected studies included author, publication year, title, participant information, context, methodology, results, themes, limitations and type of analysis used. Data are charted using a data extraction table, based on a model recommended by the JBI [59].

## 12. Risk of Bias

The Critical Appraisal Skills Programme (CASP) checklist for qualitative research is a validated tool for quality assessing qualitative research, and it is endorsed by Cochrane and the World Health Organisation [60]. The checklist is widely used and recommended for novice researchers and is known to be succinct and effective [61]. Furthermore, the checklist was developed for use in health-related research [60]. The CASP checklist tool allows the researchers to systematically evaluate published papers by looking at the reliability, relevance and conclusions drawn. The quality of the included papers was assessed using the tool by four reviewers, with each reviewer assessing 50% of the included studies. Any disagreements were resolved through discussion.

## 13. Data Synthesis

A thematic synthesis of qualitative data based on the method described by Thomas and Harden [51] was utilised to synthesise and manage the extracted data, and themes emphasising key issues and messages were created. The data synthesis process involved several key steps:

The first step involved line-by-line coding of the primary research. This meticulous process ensured that all relevant data were captured and categorised appropriately. Each line of text from the included studies was examined and assigned a code that summarised its meaning. The initial codes were then re-examined to identify similarities and relationships between them, a process termed “axial coding” [62]. During this stage, the codes were grouped into broader categories, highlighting the connections and patterns within the data. To ensure accuracy and comprehensiveness, the codes were reassessed. This step involved reviewing the codes to ensure they accurately captured the data, a process informed by guidelines from Tricco et al. [63]. This iterative process helped refine the codes and improve the reliability of the coding. Following the reassessment, the codes were organised into logical groups to develop descriptive themes. These themes provided a structured summary of the data, capturing the core messages and issues identified in the primary research. Finally, the reviewers made inferences about the experiences captured by the descriptive themes to generate analytical themes. These analytical themes went beyond mere description, offering deeper insights into the data and providing a more comprehensive understanding of the factors influencing PS among HSCPs. This systematic approach to data synthesis ensured a thorough and robust analysis of the qualitative data, allowing the reviewers to identify key themes and messages related to PS in health and social care settings.

## 14. Results

### Study Characteristics

**Figure 1:**
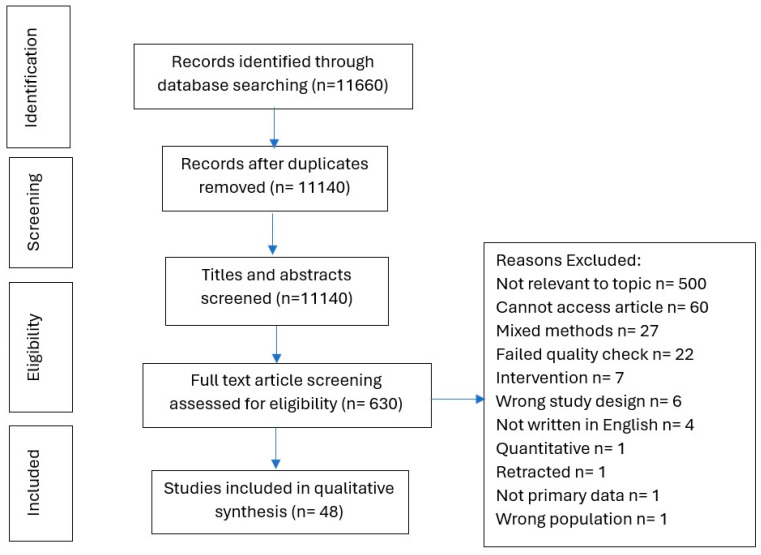
PRISMA Flow diagram.

In total, 11,660 articles were identified. After the removal of duplicates and the screening of titles and abstracts, 630 full-text articles were screened. 48 papers were included in the review. Full details of the search results and the reasons for exclusion are shown in the PRISMA diagram (Figure 1). For full study characteristics, see Table 2. The included studies were published between 2001 and 2023. Sample sizes ranged between 7 and 1636 participants: the total number of participants was 4139.

## 15. Quality Assessment

The included studies’ methodological quality was judged as being of a high standard overall. However, 37% of the studies did not explicitly state how the risk of researcher bias was minimised or how the relationship between the researcher and the participants had been considered. For further details on the quality assessment, please refer to Table 3.

## 16. Thematic Synthesis

**Figure 2:**
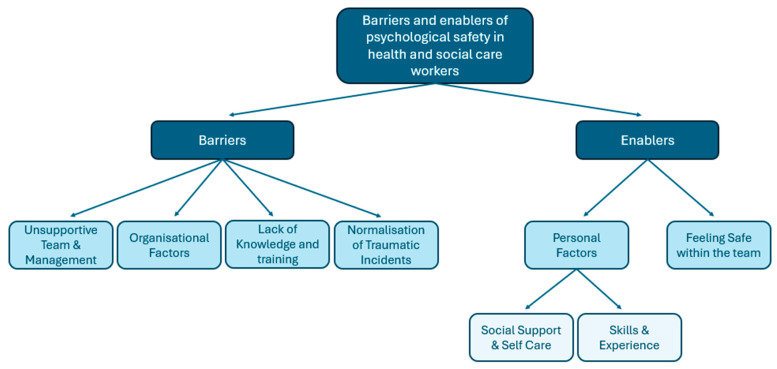
Thematic map showing the barriers and enablers of psychological safety identified in the review.

The aim of this thematic synthesis was to identify, collate and thematically synthesise the qualitative literature to identify the barriers and enablers of PS as experienced by HSCPs across diverse health and social care settings. This section presents findings of the thematic synthesis focusing on the experiences of PS among HSCPs, categorised into positive and negative experiences. Seven analytical themes were derived from the data. Table 4 presents the themes and illustrative quotes. A diagrammatic thematic map (Figure 2) of the enabler and barrier themes identified [111] is also included.

## 17. Enablers of Psychological Safety

Enablers of PS among HSCPs were identified through two main themes: ‘Personal Factors’ and ‘Feeling Safe Within the Team’.

## 18. Personal Factors

Personal factors, divided into the subthemes ‘Skills and Experience’ and ‘Social Support and Self-Care’, can promote positive experiences of PS in HSCPs by encouraging speaking up, lowering stress, and increasing confidence in the workplace.

### 18.1. Skills and Experience

Skills such as emotional intelligence, resilience, and de-escalation were found to enhance psychological safety by equipping HSCPs with the ability to cope with workplace challenges and stress [68]. Additionally, confidence and experience contributed to a sense of security in their roles [81,88,96,99]. Training, adequate resources, and a supportive community were highlighted as crucial for staff wellbeing, fostering a sense of competence and professional growth which was seen as essential for job satisfaction [66,67,75,77,83,94,102,107].

### 18.2. Social Support and Self-Care

Social support networks, engagement in self-care activities like exercise and meditation, and seeking professional help when needed were identified as promoting PS [69,70,74,79,87,88,96,99,100]. During the COVID-19 pandemic, external support proved essential for coping, underscoring the role of support beyond the workplace [85,91].

## 19. Feeling Safe Within the Team

Peer support and feeling valued within the team were critical factors in fostering psychological safety [68,86,87,88,96,99,106]. Support from colleagues, both formal and informal, helped alleviate stress and build resilience [72,105]. Positive peer relationships helped to foster a feeling of comradery which helped prevent feelings of isolation [67,70]. Clear communication and a supportive team culture encouraged staff to speak up and share ideas without fear of judgement [87,97,105]. Supervisory support during stressful times further enhanced feelings of safety and support [103,107].

## 20. Barriers to Psychological Safety

Barriers impacting PS were categorised into four themes: ‘Normalisation of Traumatic Incidents’, ‘Unsupportive Team and Management’, ‘Organisational Factors’, and ‘Lack of Knowledge and Training’.

## 21. Normalisation of Traumatic Incidents

The normalisation of workplace trauma, violence, and injuries discouraged HSCPs from reporting incidents or admitting mistakes due to fear of judgement or a culture of silence [71,78,82,84,93,95,109,110]. The normalisation of workplace trauma can lead to staff feeling vulnerable and isolated due to fear or lack of support from their organisation [78,83,86,109]. This environment led to psychological distress and physical symptoms of stress among staff, which contributes to burnout [69,79,86,87,90,99,100,109].

## 22. Unsupportive Team and Management

A negative team culture and hierarchical structures within organisations hindered PS by creating barriers to open communication and support [65,69,82,86,90,93,109]. Lack of management support and poor communication exacerbated stress and dissatisfaction among staff [97,104,105]. Feeling unsafe or scared to speak to management feeds into a perceived lack of support, which affects morale, which impacts feelings of psychological safety [78,93].

## 23. Organisational Factors

Issues such as understaffing, excessive workload, and inadequate resources contributed significantly to psychological distress and burnout among HSCPs [64,65,69,78,82,84,96,98,101,104]. During the COVID-19 pandemic, insufficient personal protective equipment (PPE) and rapidly changing protocols further exacerbated stress levels and contributed to HSCP feeling concerned for themselves, their colleagues and patients [31,67,70,75,89,91,94,102,107,108].

## 24. Lack of Knowledge and Training

Lack of training and opportunities for professional development can act as a hindrance for PS, as feelings of inadequacy and lack of growth can contribute to experiencing stress and anxiety and negatively impact job satisfaction [83,92,100]. Insufficient training and knowledge, particularly highlighted during crises like the COVID-19 pandemic, contributed to increased anxiety and uncertainty among HSCPs [31,75,91,94,108]. The lack of preparedness and professional support further diminished PS during such unprecedented events [81].

## 25. Discussion

This scoping review identifies key factors that influence PS among HSCPs, highlighting both enablers and barriers. Six main factors that affect HSCPs experiences of PS were identified. Factors identified to enable experiences of PS were ‘feeling safe within the team’ and ‘personal factors’ with the subthemes ‘skills and experience’ and ‘social support and self-care’. These enable experiences of PS by increasing confidence and encouraging speaking up and asking for help. The four factors which were identified as contributing towards barriers of psychological safety were: ‘Normalisation of Traumatic Incidents’, ‘Organisational Factors’, ‘Lack of Knowledge and Training’, and ‘Unsupportive Team and Management’.

These findings suggest that interventions aimed at improving PS should focus on enhancing leadership practices, fostering team cohesion, promoting a supportive organisational culture, and providing adequate support systems. Addressing these areas can help create a safer and more supportive environment for HSCPs, ultimately benefiting both professionals and patients. The results also point to significant gaps in the current evidence base, particularly the need for more research on interventions that can effectively enhance PS in diverse healthcare settings. Future research should explore these areas to develop evidence-based strategies that can be widely implemented. By understanding what helps and hinders PS, health and social care organisations can take informed steps to improve the working conditions for HSCPs, contributing to better mental health outcomes, job satisfaction, and patient care quality [9].

This review produced important new information on PS as experienced by HSCPs. It offered insights into the conceptual understandings of PS, characteristics of participants, study design and methodology, and factors that enabled or acted as barriers to PS. Additionally, it highlighted challenges and gaps in the existing evidence base and provided recommendations for key areas for future research and practice. Research is needed to explore the specific factors influencing PS that were under-represented or not addressed in the existing literature. This includes examining the role of organisational culture, leadership styles, trauma-informed practices and team dynamics in fostering or hindering PS [112]. Longitudinal studies could provide deeper insights into the long-term impacts of PS on HSCPs’ mental health, job satisfaction, and patient care outcomes. Such studies could help in understanding how PS evolves over time and the sustained effects of interventions aimed at improving PS [36]. Developing and testing interventions designed to enhance PS in various health and social care settings is crucial. Future research could focus on developing evidence-based strategies and tools to enhance PS and assessing their effectiveness through controlled trials. Additionally, studies should aim to capture psychophysiological data, such as heart rate variability, to gain deeper insights into the physiological correlates of PS [38]. Combining these objective measures with subjective self-reports using validated psychological scales would provide a more comprehensive understanding of HSCPs experiences of PS [33,39].

Expanding research to include diverse health and social care settings and populations will help ensure the findings are more generalisable. Studies should consider different healthcare systems, geographic regions, and socio-cultural contexts to understand how PS varies and what tailored approaches may be needed. Engaging in multidisciplinary research that combines insights from psychology, organisational behaviour, healthcare management, and other relevant fields can provide a more comprehensive understanding of PS. Collaborative efforts can enhance the development of holistic interventions and policies. The findings of this scoping review will help inform the development of impactful resources and build an evidence base on PS in health and social care settings for HSCPs, academics, policymakers, and statutory and third-sector agencies. The aim is to raise awareness and improve research in this area, ultimately leading to developing training programmes for HSCPs and leaders focused on fostering PS, communication skills, and team building; informing policies that prioritise PS in health and social care organisations, ensuring that structures and practices support a safe and supportive working environment; and guiding resource allocation to support initiatives aimed at improving PS, including mental health support services, team development activities, and organisational change efforts. Lastly, the review targets a broad scope of disciplines concerning PS, providing the opportunity to elicit more generalisable findings that can directly inform practice and policy decisions within these disciplines. By addressing the gaps and building upon the findings of this review, future research can contribute significantly to the enhancement of PS, ultimately benefiting both HSCPs and the patients they serve.

## 26. Limitations of the Review

A key limitation of this scoping review is the exclusion of grey literature due to time constraints and the overwhelming volume of results retrieved through the formal database search strategy. While this decision was necessary to ensure the feasibility and timely completion of the review, it does introduce the potential for publication bias [42,113]. Grey literature often includes valuable research from policy reports, theses, conference proceedings, and government publications, which may contain negative or null findings not found in peer-reviewed journals. As such, our findings may reflect a skewed perspective that over-represents successful interventions or positive outcomes since these are more likely to be published. Additionally, language limitations and access barriers further constrained the evidence base. Only studies published in English were included, which inevitably omits relevant insights from non-English-speaking countries. This is particularly important in a review focused on psychological safety in health and social care, where cultural and systemic differences between countries may significantly influence how PS is understood and experienced. Several potentially eligible studies could not be reviewed in full due to paywall restrictions or access issues, which also means our thematic synthesis is based only on studies we could obtain in full. These limitations may reduce the diversity and inclusivity of our findings and restrict their generalisability to global settings. Future reviews may wish to address these limitations by allowing more time for sourcing grey literature, using translation tools or multilingual review teams, and leveraging open access networks and institutional subscriptions to retrieve a broader range of studies. Doing so would help build a more comprehensive and representative picture of PS across international health and social care contexts.

## 27. Conclusions

This scoping review details the thematic synthesis of primary qualitative research exploring HSCPs’ perspectives and experiences of PS in their work settings. The review identifies key factors that facilitate or hinder PS and highlights significant gaps in the current evidence base. Recognising what helps and hinders PS in health and social care settings can inform the design and implementation of interventions aimed at fostering environments that enhance PS for both HSCPs and patients in their care. The review produced important new information on PS as experienced by HSCPs, including conceptual understandings of PS, characteristics of participants, study designs, and methodological approaches. It identified factors that enable PS, such as supportive leadership, team cohesion, positive organisational culture, and professional development opportunities. Conversely, barriers such as hierarchical structures, excessive workload, fear of reprisal, and lack of support systems were also highlighted. Significant gaps in the literature were identified, including the need for more intervention studies, research in diverse health and social care settings, longitudinal studies, consideration of socio-cultural contexts, and quantitative measures to complement qualitative findings. Future work and subsequent research could build upon the findings of this scoping review. The review will help inform the development of impactful resources and build an evidence base on PS in health and social care settings for HSCPs, academics, policymakers, and statutory and third-sector agencies. The aim is to raise awareness and improve research in this area, leading to the development of evidence-based strategies that can be widely implemented. By addressing these gaps and focusing on the identified enablers and barriers, health and social care organisations can take informed steps to improve working conditions for HSCPs, contributing to better mental health outcomes, job satisfaction, trauma-informed practices and improved patient care quality. The review will be targeted across a broad scope of disciplines concerning PS, providing the opportunity to elicit more generalisable findings that can directly inform practice and policy decisions within these disciplines.

## Figures and Tables

**Table 1:** Search strategy.

SPIDER Tool	Search Terms
S	“health care worker*” OR “social care worker” OR “welfare worker” OR “physician*” OR Nurs* OR doctor* OR “Medic” OR “social worker*” OR “care worker*” OR “support worker*” OR “occupational therapist*” OR “psychologist*” OR “health and social care” OR “midwi*”
P of I	“psychological safety” or “interpersonal risk*” OR “team*” OR “polyvagal theory” OR “occupational wellbeing” OR “workplace wellbeing” OR “workplace mental health” OR “occupational mental health” OR “trauma*” OR “work culture” OR “workplace culture” OR “physical pain” OR “workplace safety” OR “moral distress”
D	“interview* OR “focus group*” OR obser* OR ethnography OR “thematic analysis”
E	“experience*” OR “opinion*” OR outcome*” OR “satisfaction”
R	“qualitative*” OR “mixed methods”

Note: Obser* will be removed from the search for Web of Science.

**Table 2:** Table of study characteristics.

Author	Year	Aim	Occupation	Number of Participants	Sample Demographics	Data Collection Method	Method of Data Analysis
Addo et al. [64]	2020	To explore and understand moral distress from the perspective of and as experienced by midwives.	Midwives	8	35–45 years old. Experience ranging from 5 to 15+ years. Christian and Islamic faith.	Interviews	IPA
Alilu et al. [65]	2016	To identify and describe the challenges and reasons why Iranian nurses leave their profession.	Nurses	16	14 women, 2 men. Age range 24–47, 14 with a baccalaureate degree, 2 with a master’s degree, 2–15 years of nursing experience.	Interviews	Content Analysis
Alwesmi et al. [66]	2022	To explore the experiences of nurses whose patients were diagnosed with and died of COVID-19 and how this affected their wellbeing.	Nurses	7	All female, aged 25, 31, 33, 38, 44, 47, and 58. Filipino, African, Indian and Saudi. Three had less than 10 years experience; the rest had more than 15 years.	Interviews	Thematic Analysis
Appleton et al. [67]	2023	To explore how psychological well-being is maintained by healthcare professionals (HCPs) employed in a cancer setting during the COVID-19 pandemic.	Nurses; Consultants; Radiographers; AHPs (Non-Radiographers) and Support Staff (Cancer Support Workers, Healthcare Assistants)	102	83% Female, 73% full time, age range: 21–60.	Diaries and interviews	IPA
Beattie et al. [68]	2018	To examine the neurobiological response experienced by healthcare workers when exposed to workplace violence perpetrated by consumers, to inform future training and self-care strategies for staff wellbeing.	Healthcare Providers	99	Managers (n = 45, 45.5%), departmental directors (n = 21, 21.2%), OHS staff (n = 26, 26.3%), registered nurses (n = 4, 4.1%) and educators (n = 3, 3.1%). The RNs worked in the ED and the urgent care centres of two different medium regional hospitals. The majority were female (n = 69, 69.7%)	Interviews	Thematic Analysis
Beng et al. [69]	2015	Exploring the experiences of stress in palliative care providers of University Malaya Medical Centre in Malaysia.	Palliative Care Providers	20	Male (n = 2), Females (n = 18); 7 younger than 30, 8 aged 30–39, and 5 older than 40; 7 single and 13 married; 10 participants were Malay, 7 were Chinese, and 3 were Indian.	Interviews	Thematic Analysis
Blanco et al. [70]	2023	The purpose of this study was to understand the struggles and growth opportunities of NGNs entering the field of nursing during the COVID-19.	New Graduate Nurses (NGN)	40	The majority of participants were female (n = 11, 84.6%), single (n = 9, 69.2%), and nonparents (n = 9, 69.2%). Over half of the sample identified as Hispanic (n = 7, 53.8%); the remaining participants identified as non-Hispanic White (n = 5, 38.5%) and non-Hispanic Black (n = 1, 7.7%).	Focus Groups	Content Analysis
Cankaya et al. [71]	2021	To investigate in detail the traumatic birth experiences of midwives in the delivery rooms and their attitudes, reactions, and coping strategies.	Midwives	29	The mean age of the midwives (n = 29) who participated in the study was 35.37 years, and the mean number of children they had was 1.24.	Interviews	Content and Thematic Analysis
Catalan et al. [72]	2022	The study’s aims were to identify traumatic events experienced by the participants and describe ways used to cope with them, as well as to explore longer-term implications within the stories, including any indications of positive change and growth.	Healthcare Workers and Charity Volunteers	22	14 white males, 5 white females, 1 African/Afro- Caribbean female, and 2 Southeast Asian/Indian females.	Interviews	Thematic Analysis
Chen et al. [73]	2023	To explore the enablers and barriers of career satisfaction among Australian OHTs and the reasons for pursuing career changes.	Oral Health Therapists	21	Sex: Females: 15 Males: 6 Status: Currently practising: 20 Formerly registered: 1 Practice settings: Private clinical practice: 14 Public clinical practice: 7 Educational background: bachelor’s degree in OHT: 19 Diploma/Certificate in OHT: 2 Additional qualifications at bachelor’s level or below: 9	Interviews	Thematic Analysis
Cramond et al. [74]	2019	To explore the experiences of clinical psychologists working in palliative care with adults with cancer and gain an understanding of the impact of this work and how they manage it.	Clinical Psychologists	12	3 men, 9 women. Minimum 12 months experience, qualified for 3 to 26 years.	Interviews	IPA
Craw et al. [75]	2022	To investigate how nurses coped with stress while treating COVID-19 patients during the pandemic.	Nurses	15	15 females aged 26 to 62.	Interviews	Inductive Thematic Analysis
Dennis et al. [76]	2023	To elicit the nature and sources of workplace emotional distress in an international sample of intensivists.	Doctors	19	Australia (n = 13) and Israel (n = 6). The majority of respondents were male (n = 15).	Interview	Thematic Analysis
Emmarco et al. [77]	2023	To evaluate the experiences of COVID-19 in the nursing cohort.	Nurses	14	The median age of participants was 28 and ranged from 22 to 46. The median number of years working as an RN was 4.25.	Interviews.	
Ezeobele et al. [78]	2021	To explore mental health staff perspectives on assaults by psychiatric patients.	Mental Health Staff: Nurses, Physicians, Physical Therapists, and Social Service Staff	120	22–63 years old, mean age = 32.4, 46 males, 74 females, college credits to doctorate level training.	Qualitative Survey	Transcendental Phenomenology Data Analysis
Fairman et al. [79]	2014	To examine the personal and professional impact of patient suicides among hospice clinical staff, the coping strategies used by this group, and their recommendations for staff support after a patient suicide.	Clinical Staff: Nurses, Social Workers, Primary Providers, Social Counsellors, Licenced Vocational Nurses and Others	186	Participants were predominately female (78%), and the average age was 52 years (range, 28–72). Respondents included nurses (39%), social workers (20%), primary providers (physicians or nurse practitioners, 14%), spiritual counsellors (12%), licenced vocational nurses (7%), and others. On average, participants reported 21 years of clinical experience (range, 3–50) and 11 years of practice in hospice (range, 1–27).	Qualitative Survey	Coding Consensus, Co-occurrence, and Comparison
Galuska et al. [80]	2018	To add to our understanding of meaning and joy in nursing.	Registered Nurses	27	The participants included 20 females and 7 males, ranging in age from 31 to more than 60 years, with 65% older than 50 years. Nurses’ practice experience ranged from 2 to more than 40 years.	Interviews	Thematic Analysis
Grailey et al. [31]	2021	To investigate the presence of perceived stressors, psychological safety, and teamwork in healthcare professionals.	Nurses, Doctors, and Physiotherapists	49	Thirty-nine participants in this subgroup were critical care staff: 24 nurses, 9 doctors and 6 physiotherapists. 10 recruited from the emergency department: 2 nurses, 8 doctors.	Interviews	Thematic Analysis
Griffiths et al. [81]	2014	To explore doctors, nurses, and allied health professionals’ perceptions of their preparation to care for confused older patients on general hospital wards.	Senior Specialists, Doctors, Nurses, Healthcare Assistants, Occupational Therapists, Physiotherapists	60	Male (n = 12), Female (n = 48); 44 participants were White British; Mean ages: specialists = 44.4, doctors = 27.6, nurses = 38.2, healthcare assistants = 40.8, occupational therapists = 28.8, physiotherapists = 29.8.	Interviews	Thematic Analysis
Ham et al. [82]	2021	To explore psychiatric nurses’ and other psychiatric workers’ understanding of trauma in the context of their relationships with the people they care for and the effects on their mental health.	Psychiatric Nurses and Other Psychiatric Workers	30	Most were women (n = 26, 87%), aged 40 years or older (n = 22, 73%). The largest professional group was nurses (n = 14, 47%), followed by allied health professionals (n = 12, 40%), and most had more than 10 years of experience working in the mental health field (n = 20, 67%).	Qualitative Survey	Thematic Inductive Analysis
Jakobsson Larsson et al. [83]	2023	To describe what registered nurses’ experience to be important to job satisfaction in nursing home settings.	Registered Nurses	16	Employer: Public non-profit: 8 Private for-profit: 8 Age: 25–35: 6 36–45: 4 46–55: 4 56–65: 2	Interviews	Systematic Text Condensation, a method for thematic analysis of qualitative data
Jeong et al. [84]	2016	To survey the psychological discomfort and coping processes of healthcare workers that suffered needle stick injuries.	Doctors, Nurses, Clinical Pathologists, Sanitation Workers, Medical Engineers	15	Males (n = 5), females (n = 10); 3 participants were doctors, 8 were nurses, 2 were clinical pathologists, 1 was a sanitation worker, and 1 was a medical engineer. Workplace experience ranges from 2 months to 17 years.	Interviews	Content Analysis
Jiang et al. [85]	2022	To explore the process and influencing factors of post-traumatic growth among emergency nurses infected with COVID-19.	Nurses	13	3 male, 10 female (76.92%), 84.61% single, average length of employment 3.92 years.	Interviews	Interpretative Phenomenological Approach
Ketelaar et al. [86]	2015	To investigate Dutch novice nurses’ experiences and needs regarding occupational health support to prevent work-related health problems and promote wellbeing.	Nursing Students and Newly Qualified Nurses	14	Participating nursing students were aged 23–45 (mean = 31, SD = 8.3), while participating newly qualified nurses were aged 23–40 (mean = 29, SD = 6.1).	Interviews	Grounded Theory Approach
Lases et al. [87]	2018	To investigate residents’ experiences of wellbeing in relation to their professional life.	Residents	13	6 women and 7 men; ages ranged from 26 to 34 years, and their years of residency from first to fourth.	Interviews	Thematic Network Analysis
Lewis [88]	2017	To investigate the affective, interactional, and meaning-related responses of NICU nurses caring for dying newborns.	Nurses	36	35 of the 36 participants were all white females aged 25–65 years. One participant did not complete demographic details to maintain anonymity.	Online Survey	Reismann’s Thematic Narrative Analysis
Lewis O’Connor et al. [89]	2023	The purpose of this study was to describe the experience of clinical nurses and to assess their professional quality of life after the first phase of the pandemic.	Clinical Nurses and Nurse Leaders	278	unknown	Surveys and Interviews	Inductive Thematic Analysis
McNamara et al. [90]	2018	To explore the attitudes and responses that Irish obstetricians have following direct involvement with an intrapartum foetal death.	Obstetricians and Registrars	10	5 were consultant obstetricians and 5 were registrars; 4 were exposed to intrapartum death (IPD) once, 4 were exposed twice, and 2 were exposed 3+ times.	Interviews	Interpretative Phenomenological Analysis
Mediavilla et al. [91]	2022	To describe mental health problems among frontline HCWs, investigate their associations with determinants and outcomes, and consider the implications for the design and implementation of mental health programmes in Spain.	HCWs (Doctors, Nurses, Nursing Assistants, Porters, Psychologists, Administrative Staff, and Unit Managers)	75	Participants were above the age of 18 and balanced in age and gender. 75 participants during phase 1 and 22 participants during phase 2.	Interview	Thematic Analysis
Michael & Jenkins [92]	2001	To describe the range and experience of traumatic events reported by perioperative nurses.	Nurses	233	96.6% female, mean age = 38 for males, 41 for females.	Qualitative Survey	Qualitative Content Analysis
Pavithra et al. [93]	2022	To develop an understanding of hospital staff experiences of unprofessional behaviours and their impact on staff and patients.	Doctors, Nurses, Midwives, Social and Welfare Workers, Management, Support Service Workers	1636	77.9% female, 19.7% male, and 2.4% other/non specified.	Qualitative Survey	Directed Content Analysis
Peng et al. [94]	2021	To explore the experiences of frontline nurses who had been fighting against the COVID-19 infection since the outbreak.	Nurses	20	Twenty nurses, 5 of whom are supervisor nurses, aged 24 to 43 years old. 3 with diploma degrees, 16 with bachelor’s degrees, and 1 with a postgraduate degree.	Interviews	Thematic Analysis
Powell et al. [95]	2022	To investigate the experience of the emergency nurses assaulted by a patient or visitors.	Emergency Nurses	11	Eleven experienced emergency registered nurses from 3 mid-Atlantic hospitals participated in the study.	Interviews	Thematic Analysis
Ramalisa et al. [96]	2018	To identify the specific barriers and facilitators of psychological safety in primary care teams.	General Practitioners, Practice Managers, Partners, Healthcare Assistants and Nurses	20	Anonymised.	Interviews	Thematic Analysis
Remtulla et al. [97]	2021	To investigate and discuss methods to improve nurses’ resilience in a work environment with involuntary mental healthcare users.	Nurses	24	Psychiatric nurses, the majority of whom were female.	Written Narrative	Deductive Thematic Analysis
Sanchez-Munoz et al. [98]	2023	to describe and understand the experiences of nurses during their training process in the specialty of Family and Community Nursing in Spain.	Family and Community Nurses	16	Female, mean age: 29.88 years (SD = 6.2).	Interviews and a focus group	Thematic Analysis
Siffleet et al. [99]	2015	To explore the perspective of experienced intensive care nurses regarding the maintenance of their emotional wellbeing.	Nurses	15	Fifteen registered nurses, with a mean age of 39.4 (26–50) years, were interviewed. The length of time working in ICU ranged from 3 to 25 years, with a mean of 13 years, and most (n = 12) were female. All 15 nurses indicated their intention to remain in ICU.	Interviews	Thematic Analysis
Smith & Hanna [100]	2021	To investigate the impacts of VT on the wellbeing of social workers and what, if any, self-care strategies social workers utilised.	Social Workers	4	Participant experience ranged from 5 to 18 years. 2 participants were New Zealand European, 1 was New Zealand with Maori heritage and 1 was Southeast Asian.	Interviews	Thematic Analysis
Sobekwa & Arunachallam [101]	2015	To explore and describe the lived experiences of nurses who care for mental healthcare users in an acute admission unit at a psychiatric hospital.	Nurses	12	3 men, 9 women, average length of qualification: 12.9 years (3–26).	Interviews	Interpretative Phenomenological Analysis
Sun et al. [102]	2020	To explore the psychology of nurses caring for COVID-19 patients.	Nurses	20	Males (n = 3), females (n = 17); age range of 25–49 years, with an average age of 30.60 ± 6.12. Working experience ranged from 1 to 28 years, with an average of 5.85 ± 6.43. All nurses possessed a bachelor’s degree. There were 17 general nurses and 3 head nurses.	Interviews	Phenomenological Analysis
Thude et al. [103]	2021	To understand how Danish nurses coped with the fast, comprehensive organisational changes in their workplace in order to identify barriers to and facilitators for organisations ensuring the best possible conditions for nurses to meet these challenges.	Nurses	23	All 23 interviewed nurses were female, with a mean age of 41 (26–54 years old) and a mean of 13 years of experience as a nurse (0.5–27).	Naive Reading of the Text	Thematic Analysis
O’Toole et al. [104]	2021	To assess factors related to training and practice that posed a threat to physician wellbeing or increased burnout and to describe suggestions from fellows on how training programmes can improve physician wellbeing.	Doctors	427	60% men, 59% white, 76% married, and 41% had caregiver responsibilities. 33% screened positive for burnout, and 41% screened positive for depressive symptoms.	Qualitative Survey	Inductive Qualitative Content Analysis
Voogt et al. [105]	2020	To explore what helps residents speak up about organisational barriers and opportunities to improve the quality of their work and what hinders them from doing so.	Medical Residents	27	19 (70%) female, mean age was 31 years (SD = 4, range 26–48), mean postgraduate year was 2.8 (SD = 1, range 1–6).	Interviews	QUAGOL
Walker et al. [106]	2022	To examine protective factors that promote wellbeing and professional fulfilment in surgeons.	Surgeons	32	Males (n = 20), females (n = 12); clinical full-time equivalent (FTE) ranged from 0% to 90% (median = 65%). Age ranged from 41 to 72 years. 84.3% (n = 27) were Caucasian.	Interviews	Abductive Exploratory Analysis
Warren et al. [107]	2021	To understand the experiences of trainees working in a large intensive care unit during the first wave of COVID-19 from an educational and operational perspective in order to highlight what worked and what could be improved.	Trainees in Anaesthesia and Intensive Care	40	Not specified	Interviews	Thematic Analysis
Welsh et al. [108]	2021	To investigate the impact of COVID-19 on emergency physicians’ emotional experiences and the specific coping strategies that physicians employed throughout the pandemic.	Physicians	26	Metro Boston Region—10 males, 5 females; 13 white, 1 black, 1 Asian, 1 Hispanic, 14 not Hispanic; 6 hold a leadership position. New York City—4 male, 7 females; 6 white, 1 black, 3 Asian, other (middle eastern) 1, 2 Hispanic, 9 not Hispanic; 4 hold a leadership position.	Interviews	Comparative Analysis
Xu et al. [109]	2023	to elicit labour and delivery clinician perspectives on the impact of exposure to traumatic births on their professional quality of life.	L&D Clinicians, Including Attending or Resident Physicians, Advanced Practice Nurses, Including Certified Nurse Midwives, and Nurses	165	The majority of respondents were female, White physicians, with 13.2 mean years of L&D experience.	Anonymous online questionnaire and phone interviews	Grounded Theory
Zheigami et al. [110]	2021	To investigate the effects of sexual harassment in the workplace on nurses.	Nurses	22	18 female, 4 male. 25–51 years old, work experience ranging from 2 to 28 years.	Interviews	Content Analysis

**Table 3:** Quality Assessment.

Study Reference	Q1	Q2	Q3	Q4	Q5	Q6	Q7	Q8	Q9	Q10
Addo at al. [64]	Y	Y	Y	Y	Y	N	N	Y	Y	Y
Alilu et al. [65]	Y	Y	Y	Y	Y	N	Y	Y	Y	Y
Alwesmi et al. [66]	Y	Y	Y	Y	Y	Y	Y	Y	Y	Y
Appleton et al. [67]	Y	Y	Y	Y	Y	N	Y	Y	Y	Y
Beattie et al. [68]	Y	Y	Y	Y	Y	N	Y	Y	Y	Y
Beng et al. [69]	Y	Y	Y	Y	Y	N	Y	Y	Y	Y
Blanco et al. [70]	Y	Y	Y	Y	Y	Y	Y	Y	Y	Y
Cankaya et al. [71]	Y	Y	Y	Y	Y	Y	Y	Y	Y	Y
Catalan et al. [72]	Y	Y	Y	Y	Y	Y	Y	Y	Y	Y
Chen et al. [73]	Y	Y	Y	Y	Y	Y	Y	Y	Y	Y
Cramond et al. [74]	Y	Y	Y	Y	Y	Y	Y	Y	Y	Y
Craw et al. [75]	Y	Y	Y	Y	Y	Y	Y	Y	Y	Y
Dennis et al. [76]	Y	Y	Y	Y	Y	Y	Y	Y	Y	Y
Emmarco et al. [77]	Y	Y	Y	Y	Y	N	Y	Y	Y	Y
Ezeobele et al. [78]	Y	Y	Y	Y	Y	Y	Y	Y	Y	Y
Fairman et al. [79]	Y	Y	Y	Y	Y	Y	Y	Y	Y	Y
Galuska et al. [80]	Y	Y	Y	Y	Y	N	Y	Y	Y	Y
Grailey et al. [31]	Y	Y	Y	Y	Y	Y	Y	Y	Y	Y
Griffiths et al. [81]	Y	Y	Y	Y	Y	N	Y	Y	Y	Y
Ham et al. [82]	Y	Y	Y	Y	Y	N	Y	Y	Y	Y
Jakobsson Larsson et al. [83]	Y	Y	Y	Y	Y	Y	Y	Y	Y	Y
Jeong et al. [84]	Y	Y	Y	Y	Y	Y	Y	Y	Y	Y
Jiang et al. [85]	Y	Y	Y	Y	Y	Y	Y	Y	Y	Y
Ketelaar et al. [86]	Y	Y	Y	Y	Y	N	Y	Y	Y	Y
Lases et al. [87]	Y	Y	Y	Y	Y	Y	Y	Y	Y	Y
Lewis & Ahern [88]	Y	Y	Y	Y	Y	N	Y	Y	Y	Y
Lewis O’Connor et al. [89]	Y	Y	Y	Y	Y	N	Y	Y	Y	Y
McNamara et al. [90]	Y	Y	Y	Y	Y	Y	Y	Y	Y	Y
Mediavilla et al. [91]	Y	Y	Y	Y	Y	Y	Y	Y	Y	Y
Michael & Jenkins [92]	Y	Y	Y	Y	Y	N	Y	Y	Y	Y
O’Toole et al. [104]	Y	Y	Y	Y	Y	Y	Y	Y	Y	Y
Pavithra et al. [93]	Y	Y	Y	Y	Y	N	Y	Y	Y	Y
Peng et al. [94]	Y	Y	Y	Y	Y	Y	Y	Y	Y	Y
Powell et al. [95]	Y	Y	Y	Y	Y	Y	Y	Y	Y	Y
Ramalisa et al. [96]	Y	Y	Y	Y	Y	Y	Y	Y	Y	Y
Remtulla et al. [97]	Y	Y	Y	Y	Y	Y	Y	Y	Y	Y
Sanchez-Munoz et al. [98]	Y	Y	Y	Y	Y	N	Y	Y	Y	Y
Siffleet et al. [99]	Y	Y	Y	Y	Y	N	Y	Y	Y	Y
Smith & Hanna [100]	Y	Y	Y	Y	Y	N	Y	Y	Y	Y
Sobekwa & Arunchallam [101]	Y	Y	Y	Y	Y	Y	Y	Y	Y	Y
Sun et al. [102]	Y	Y	Y	Y	Y	Y	Y	Y	Y	Y
Thude et al. [103]	Y	Y	Y	Y	Y	Y	Y	Y	Y	Y
Voogt et al. [105]	Y	Y	Y	Y	Y	Y	Y	Y	Y	Y
Walker et al. [106]	Y	Y	Y	Y	Y	Y	Y	Y	Y	Y
Warren at al. [107]	Y	Y	Y	Y	Y	Y	Y	Y	Y	Y
Welsh et al. [108]	Y	Y	Y	Y	Y	Y	Y	Y	Y	Y
Xu et al. [109]	Y	Y	Y	Y	Y	N	Y	Y	Y	Y
Zeighami et al. [110]	Y	Y	Y	Y	Y	Y	Y	Y	Y	Y

Note: Q stands for question, in relation to the set of 10 questions in the Critical Appraisal Skills Programme (CASP) checklist tool. Y stands for Yes; the study passed this question. N stands for No; the study did not pass this question. Q1: Was there a clear statement of the aims of the research? Q2: Is a qualitative methodology appropriate? Q3: Was the research design appropriate to address the aims of the research? Q4: Was the recruitment strategy appropriate to the aims of the research? Q5: Was the data collected in a way that addressed the research issue? Q6: Has the relationship between researcher and participants been adequately considered? Q7: Have ethical issues been taken into consideration? Q8: Was the data analysis sufficiently rigorous? Q9: Is there a clear statement of findings? Q10: How valuable is the research?

**Table 4:** Summary of analytical themes and illustrative quotes.

Analytical Theme	Subtheme	Quote
Personal Factors	Skills and Experience	“I think that is something that comes with the knowledge and experience of being in that scenario…Experience definitely has a lot to do with it and I think seeing how other people handle situations. I haven’t just got seven years of experience myself; I’ve got seven years of observing other people, which definitely contributes” (Siffleet et al., 2015; p. 309) [99] “I felt like it was a good growing experience because even though growth is hard, I feel like I was able to become a lot more independent and a lot more confident in my practice and learned to appreciate how much easier the rest of our life is” (Emmarco et al., 2023; p. 270) [77].
	Social Support and Self-Care	“You must put your own needs first because you cannot care for anyone if you are not taking care of yourself first. I can only help my clients if I take care of myself first” (Smith & Hanna, 2021; p. 55) [100]
Feeling Safe Within the Team		“We don’t discuss it but each one of us sort of understand how we are suffering from the death of our patients and from the whole pandemic situation. We all feel the exhaustion, frustration, so we silently help each other do the daily work in our unit, our team work became stronger and we learned to be more sensitive to each other” (Alwesmi et al., 2022; p. 5) [66] “What teamwork means for me is looking out for everybody else that is round about me and if I see anybody struggling I will go in, I will ask them…if they needassistance…if you help them out when you are in the same situation one day it is normally reciprocated” (Siffleet et al., 2015; p. 308) [99]. “It is a pleasant feeling when there is an accessible atmosphere in residency training. This makes me more inclined to ask questions, discuss things and encourages me to reflect on former situations. It is nice to notice that the supervisors also appreciate this” (Lases et al., 2018; p. 985) [87].
Normalisation of Traumatic Incidents		“I felt shocked because my patient was doing well and then all of a sudden she’s gone” (Alwesmi et al., 2022; p. 5) [66] “Our superiors always blame us for any injury we sustained from a patient assault, label us as “unfit” for the job and the perpetrator of the assault. This dampens our spirit, causes demotivation, low morale... and for many of us, the work becomes, “a mere job” and this behavior makes us want to quit” (Ezeobele et al., 2021; p. 247) [78] “the most harmful intervention would be to ignore the situation or keep it ‘‘hush hush’’ and make it seem as if it never happened” (Fairman et al., 2014; p. 834) [79]
Unsupportive Team and Management	Team Culture	“Some of the nurses, they just don’t care. If I talk to them, they just walk away. I complain to the nurse manager, but I think they won’t change. I think that’s how they are trained, that’s how their attitude is” (Beng et al., 2015; p. 17) [69] “I feel when an event happens there is no support” (Ham et al., 2021; p. 1485) [82] “I’ve seen people bullied and confidence squashed to the point they resign as an anxious mess” (Pavithra et al., 2022; p. 7) [93]
	Hierarchical Structure	“My boss has left now—she used to hang up on me if I called in sick…Scream at me if I asked for a day off, refused to give me long service leave. I was scared of her; I wasn’t the only one. Reporting would not have worked” (Pavithra et al., 2022; p. 8–9) [93] “Many of us are very sad for the fact that physicians have such high status in the hospital, especially male doctors and we [nurse] are treated as worthless” (Alilu et al., 2016; p. 537) [65] “Sometimes we can feel the kind of separation like you feel like your input is slightly valued less than a doctor’s would be” (Remtulla et al., 2021; p. 7) [97]
Organisational Factors		“The existing protocols are not adequate or sometimes not relevant to control the spread of the infection” (Alwesmi et al., 2022; p. 5) [66] “There was just not enough of us. We wanted to make sure we were taking care of ourselves, but it was difficult because you couldn’t just leave the room” (Emmarco et al., 2023; p. 269) [77] “Here we have four staff per shift, but we are taking care of twenty patients. Sometimes the patients are very ill. Sometimes they want more attention, but we can’t help” (Beng et al., 2015; p. 19) [69]. “Almost every day we are assigned to more patients than we can care for” (Alilu et al., 2016; p. 539) [65]
Lack of Knowledge and Training		“I think employers need to be more supportive in terms of practitioner professional development. This is what I am missing. I want the training but there doesn’t seem to be much available” (Smith & Hanna, 2021; p. 56) [100]. “Some of my colleagues have years of experience and near retirement, but they are still working as staff nurses instead of being promoted to mentor our new graduates and when I see them, I can picture my own professional future without recognition or promotion” (Alilu et al., 2016; p. 538) [65]

## Data Availability

No new data were created or analysed in this study. Data sharing is not applicable to this article.

## Public Involvement Statement

No public involvement in any aspect of this research.

## Guidelines and Standards Statement

This manuscript was drafted in accordance with the Preferred Reporting Items for Systematic reviews and Meta-Analyses extension for Scoping Reviews checklist (PRISMA-ScR) for Scoping Review research.
